# Surface electromyographic (sEMG) activity of the suprahyoid and sternocleidomastoid muscles in pitch and loudness control

**DOI:** 10.3389/fphys.2023.1147795

**Published:** 2023-05-04

**Authors:** Feifan Wang, Edwin M.-L. Yiu

**Affiliations:** Voice Research Laboratory, Faculty of Education, The University of Hong Kong, Pokfulam, Hong Kong SAR, China

**Keywords:** surface electromyography, laryngeal muscles, speech, pitch, loudness

## Abstract

**Purpose:** This study set out to determine the contributions of the suprahyoid and sternocleidomastoid (SCM) muscles in changing pitch and loudness during phonation among vocally healthy populations.

**Method:** Thirty-nine participants were first recruited, and twenty-nine of them who passed the screening test (Voice Handicap Index [VHI]-10 score ≤11, auditory-perceptual voice rating score ≤2) were finally selected (mean age = 28.2 years). All participants were measured for their surface electromyographic (sEMG) activity collected from the bilateral suprahyoid and SCM muscles when producing the vowel /a/, /i/, and /u/ in natural (baseline) and at different pitch (+3, +6, -3, -6 semitones) and loudness (+5, +10, −5 dB) levels. Linear mixed-effects models were performed to determine the influencing factors on the root-mean-square percentage of maximal voluntary contraction (RMS %MVC) value of the sEMG signals.

**Results:** Compared with the baseline, a significant decrease of RMS %MVC was found in the suprahyoid muscles during overall phonations of lower pitches (−3 and −6 semitones) and loudness (−5 dB). However, no significant change was detected when producing speech at higher pitch (+3 and +6 semitones) and loudness (+5 and +10 dB) levels. Among the three vowels, /i/ demonstrated significantly higher RMS %MVC than those of /a/ and /u/. The SCM muscles, however, did not show any significant change in the RMS %MVC values among different vowels in relation to the pitch and loudness changes. When the muscles were compared across the two sides, significantly higher RMS %MVC was found in the right side of the suprahyoid (in pitch and loudness control) and SCM (in pitch control) when compared to the left side.

**Conclusions:** The suprahyoid muscle activities were significantly decreased when producing lower pitches and intensities compared to the natural baselines. The production of sustained /i/ required significantly more suprahyoid muscle activities than those of /a/ and /u/. The SCM muscles did not show much sEMG activity in any of the pitch and loudness levels, which could be used potentially as the calibration or normalization of peri-laryngeal sEMG measurement. The findings also showed a tendency for bilateral asymmetry in the use of suprahyoid and SCM muscles.

## 1 Introduction

Phonation plays an important role in human communication, which is a complex system relying on the laryngeal and peri-laryngeal muscles to change the pitch and loudness at all times (Vilkman et al., 1996). The intrinsic laryngeal muscles, which connect different anatomical parts of the larynx, were considered to control the tension of the vocal folds, hence determining the subglottic pressure, and the airflow (Hirano et al., 1969). The extrinsic laryngeal muscles bounded by the hyoid bone and divided into the suprahyoid and infrahyoid muscle groups, were more directly related to the position of the larynx (Honda et al., 1999). The suprahyoid muscles (e.g., the geniohyoid, mylohyoid, stylohyoid, and digastricus muscles) serve as the elevator that helps draw the hyoid bone upwards and lift the larynx, while the infrahyoid muscles (e.g., the sternothyroid, thyrohyoid, and the sternohyoid muscles) play the role of a depressor that pulls down the hyoid bone and lowers the larynx (Marchal, 2009).

Although in theory, an elevated hyoid bone or larynx usually correlates with an increase in pitch production (and *vice versa*, e.g., see Honda et al., 1999), researchers in practice, however, have no consensus on how different extrinsic laryngeal muscles participate or respond to changes of pitch. In the 1990s, Vilkman et al. (1996) reviewed 15 studies that investigated the contributions of extrinsic laryngeal muscles to pitch variations. They found that 1) the infrahyoid muscles received more attention (15 studies) than the suprahyoid muscles (5 studies); 2) none of the muscles showed a consistent contribution for pitch variations among different studies, or even different subjects in a certain study. It should be noted that the phonation tasks (e.g., singing, speech, intonation) and pitch ranges (e.g., extreme pitch range, two octaves) varied among these 15 studies, which made it difficult to compare and extract consistent evidence on how extrinsic laryngeal muscles contribute to pitch manipulations.

Fewer studies focused specifically on the correlations between the extrinsic laryngeal muscles and control of loudness. Hirano et al. (1967) studied specifically the function of the sternohyoid muscle (which belongs to the infrahyoid muscle group) and found an increased electrical activity following the increasing loudness at different pitch levels. However, they also noted that these muscle activation patterns differed among subjects depending on individual vocal habits or experience of vocal training. Dietrich and Verdolini Abbott (2012) indicated that the infrahyoid muscle activities were increased along with the self-perceived vocal effort under stressful conditions, while McKenna et al. (2019) reported stronger activation of the suprahyoid muscles with an increased self-perception of vocal effort.

Pitch and loudness control is generally associated with vocal effort. Hence, they were commonly used as vocal loading tasks (Fujiki and Sivasankar, 2017). The excessive vocal effort might result in an increased extrinsic laryngeal muscle tension, which was so often recognized as a possible cause and/or typical symptom of voice disorders, specifically in muscle tension dysphonia and vocal hyperfunction (Van Houtte et al., 2011). An increasing number of studies, therefore, attempted to investigate the clinical values of the extrinsic laryngeal muscles for the diagnosis of voice disorders (Khoddami et al., 2013) using surface electromyography (sEMG). Surface EMG has the advantage of being non-invasive in measuring the activities of the relatively superficial extrinsic laryngeal muscles in the peri-laryngeal area (Stepp, 2012). One would expect that the sEMG might be a useful clinical tool in identifying typical voice disorders which involve laryngeal muscle activities. However, recent reviews (Wang and Yiu, 2021; Desjardins et al., 2022) showed that this is far from the truth. There is still no consensus on how to reliably identify voice disorders using sEMG. Wang and Yiu (2021) pointed out that there is little agreement on what phonation tasks should be used in sEMG measurement for the peri-laryngeal muscles. This is probably due to little knowledge of how sEMG measurement can be affected differently by different degrees of task design, such as changes in pitch and loudness. It is therefore necessary to investigate how the control of pitch and loudness in sustained phonation tasks affects the measurement of sEMG activity in the peri-laryngeal muscles.

The sternocleidomastoid (SCM) muscles are often classified as respiratory muscles in the peri-laryngeal area that are not so important for speech production (Stepp, 2012). However, it has been found that the SCM muscles were also activated during speech (Pettersen et al., 2005), and varied with the change in loudness (Arifin et al., 2014) and pitch (Yu et al., 2016). A recent study also found a fatigue-related change in the sEMG signals of the SCM muscles after a vocal loading task (Yiu et al., 2023). Compared with other peri-laryngeal muscles, the SCM muscles have relatively large sizes suitable for sEMG measures, but whether or how they worked during pitch and loudness control in sustained phonation remains unclear so far.

A better understanding of the role that the suprahyoid and SCM muscles play in phonation would not only help to understand the physiological mechanism of the neuromuscular modulation during speech and voice production, but also shed light on the clinical pathology of voice disorders. The present study, therefore, aimed to determine the contributions of the suprahyoid and SCM muscles to pitch and loudness control. We hypothesized that the target muscles would participate in pitch and loudness control and might show different activities in changing pitch and loudness levels.

## 2 Materials and methods

### 2.1 Participants

Thirty-nine healthy adults (21 females, 18 males) aged between 20 and 78 years (*M* = 30.4 years, *SD* = 11.1 years) were recruited. All participants were current Hong Kong Chinese residents without a reported history of language, hearing, or voice disorders. None of the participants reported a prior experience with professional singing training. The present study was approved by the Human Research Ethics Committee (HREC), The University of Hong Kong (EA2001023(A)). Participants had enough time to fully read all the related information and ask any questions about this study. They had to sign a written consent before they formally took part in this study.

### 2.2 Procedure

#### 2.2.1 Participant screening

All participants, although reported no voice disorders by themselves, were further ascertained for their vocal conditions. First, each participant was asked to fill in a Chinese Voice Handicap Index-10 (VHI-10) questionnaire (Lam et al., 2006) to determine their voice conditions and how their voices affected their lives. Using the cut-off point as suggested by Arffa et al. (2012), only participants who got a VHI-10 score of 11 or less were included in the study.

These participants were then asked to produce sustained vowels /a/, /i/, and /u/ (each for 3–5 s) and read a Chinese poetry *Jingye Si* at a comfortable pitch and loudness. The Chinese poetry *Jingye Si* contained different phonemes that required diverse laryngeal behaviors (see the complementary materials) and was used to measure speech, voice, and respiratory signals (Li, 2015). Samples were recorded using the Visi-Pitch (KAY3950c, PENTAX, Montvale, NJ) and high-quality microphone (SHURE SM48, Niles, IL) with a microphone-to-mouth distance of 10 cm. Two female certified speech pathologists, who were current PhD students in voice and swallowing disorders and had experiences with voice-disordered patients, undertook an auditory-perceptual rating for each of the recordings independently. These two judges were blinded to the VHI-10 scores of each participant and made an overall judgment on each participant’s voice using an equal-appearing interval scale from 0 (no voice problem) to 10 (severe voice problem). Based on the findings of auditory-perceptual voice evaluation studies using a visual analog scale (VAS), a cut-off value of 2/10 points (usually more than 30/100 points in previous literature, e.g., see Lee et al., 2018; Simberg et al., 2000) was used to select the healthy participants to be included in the final study.

#### 2.2.2 Surface EMG (sEMG) setup

Each participant was required to sit upright on a backrest chair with four surface electrodes (single-differential Trigno™ Mini sensor, Delsys, Natick) taped on the skin surface of the neck area. To avoid a high skin-electrode impedance, all participants were informed in advance to shave the anterior neck area (if applicable) the day before the study. In addition, a 70% isopropyl alcohol wipe was used to clean the skin of the neck area before electrode placement (Stepp, 2012).

**FIGURE 1:**
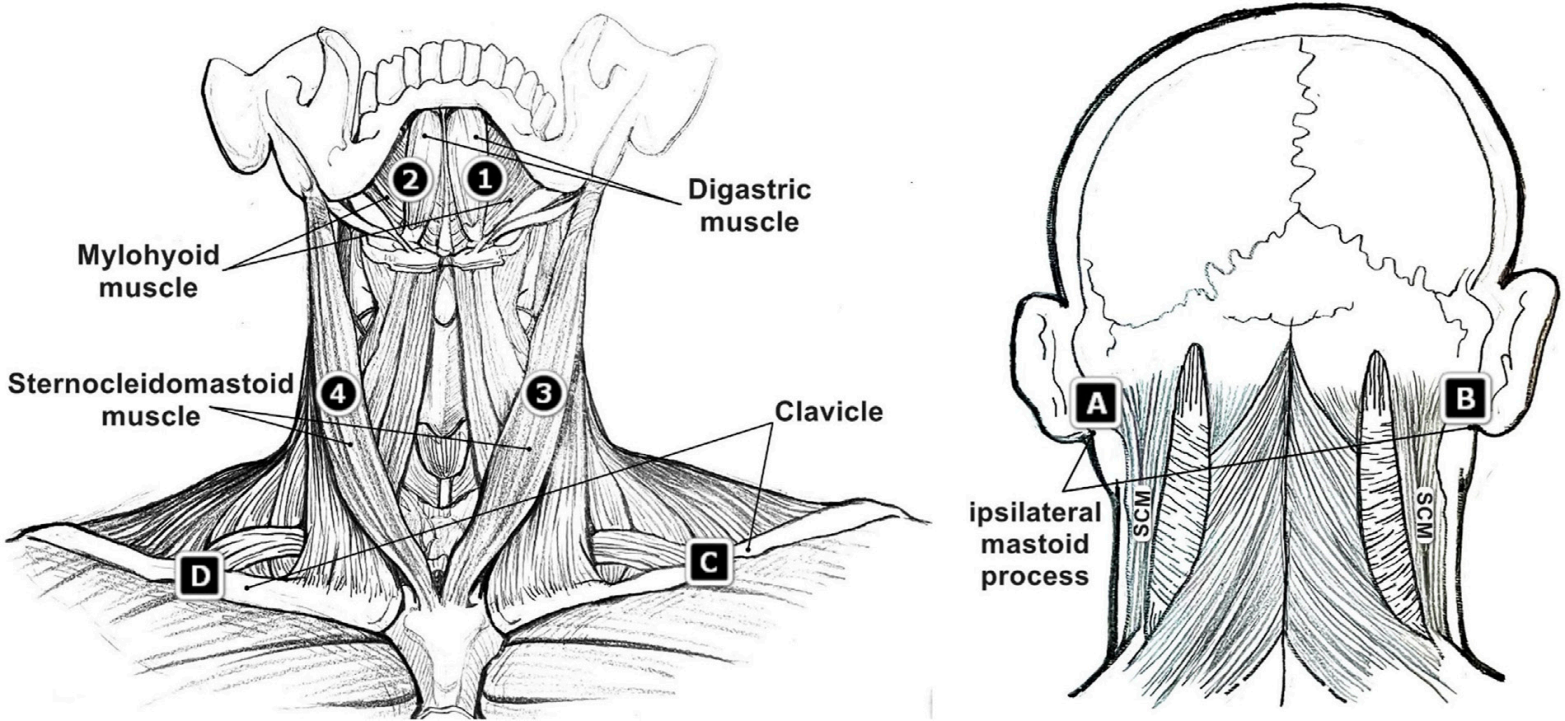
Surface electromyographic (sEMG) sensor placement (sensor 1, 2, 3, 4 and corresponding ground sensor A, B, C, D) on the region of suprahyoid muscles and sternocleidomastoid (SCM) muscles.

The placement of the sEMG electrodes was demonstrated in Figure 1, mainly targeting the bilateral suprahyoid and SCM muscles. Sensor 1 and 2 were placed symmetrically in the submandibular region, each was about 1 cm away from the midline. These two electrodes were used to detect the activation of the suprahyoid muscles involving the anterior belly of the digastric muscle, the anterior mylohyoid muscle, and the geniohyoid muscles. The potential activities of the platysma muscle, a superficial layer overlaying the suprahyoid area, were also unavoidably collected (Stepp, 2012). The associated ground sensor A and B were placed on the ipsilateral mastoid process. Sensor 3 and 4 were placed approximately at the mid-point of the left and right SCM muscles, with the corresponding ground sensor C and D on the mid-point of the left and right clavicle. The clavicle and ipsilateral mastoid process were superficial and appropriate in size for the ground sensor placement. To minimize the “cross-talk” between electrode sites, every two electrodes were confirmed to keep a distance of no less than 2 cm (Stepp, 2012).

#### 2.2.3 Recordings of the sEMG signals

Participants were first required to complete two maximal voluntary contraction (MVC) tasks. The sEMG data collected during the MVC tasks would serve as a reference for data normalizations that would ensure comparisons among individuals under different conditions (Netto and Burnett, 2006; Stepp, 2012). For the suprahyoid muscles, participants were asked to keep seated and put their chins on a stable platform (the height of the platform was adjusted to be flush with each participant’s chin) and then pressed their chins down against the platform by exerting a maximum force for about 3 s (Redenbaugh and Reich, 1989; Stepp et al., 2011). Participants were required to keep a vertically downward force without any other movement of the head and shoulders throughout the process. For the SCM muscles, participants sat with a stable body and resting arms and then performed an isometric neck flexion 90° to the left (for the MVC of the right SCM) and then to the right (for the MVC of the left SCM), each with their maximum force for about 3 s (Barbero et al.,2012).

**FIGURE 2:**
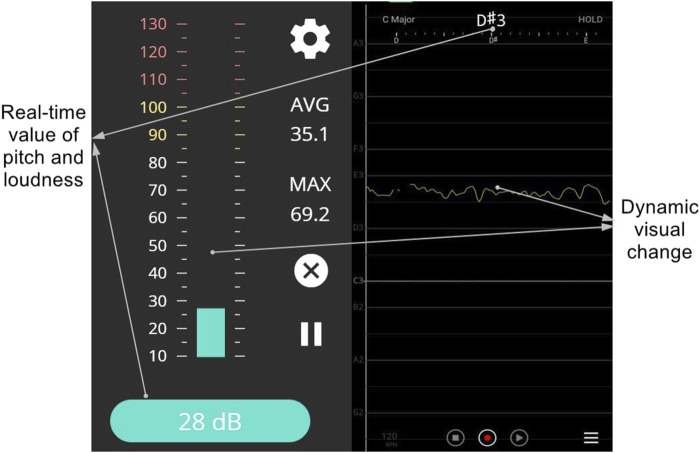
Visual feedback interfaces demonstrating real-time loudness (left, displayed with the online loudness and sound meter by Youlean) and pitch (right, displayed with the Vocal Pitch Monitor Application).

Each participant was then asked to produce a prolongation of each of the vowels /a/, /i/, and /u/ respectively for 4 seconds at a comfortable pitch and loudness level. These three vowels were selected as previous literature found that the extrinsic laryngeal muscle activities varied depending on different vowels (Faaborg-Andersen and Vennard, 1964). Real-time visual feedback (including the instantaneous values and value changes over time) of the pitch and loudness (as shown in Figure 2) was provided to each participant using a Vocal Pitch Monitor application (Tadao, 2019) and online Youlean loudness meter (Nikolic, 2020) on a Microsoft Surface Pro Pad (Microsoft, model 1796) which was set up about 20 cm away from the participant. The baseline levels of pitch and loudness for a natural phonation of /a/, /i/, and /u/ were recorded. Participants were then asked to produce the vowel /a/, /i/, and /u/ at four different pitch levels (+3 semitones, +6 semitones, −3 semitones, −6 semitones, compared with the baseline) and three different loudness levels (+5 dB, +10dB, −5dB, compared with the baseline) respectively with the assistance of the real-time visual feedback. They were given a few practices by specifically producing different pitches without producing great variations of the loudness, and *vice versa*. After the practices, participants then produced the vowel /a/, /i/, and /u/ respectively for 4 seconds at four different pitch levels and three different loudness levels in a randomized order for sEMG recording. The real-time visual feedback of the pitch and loudness levels was always shown to each participant. An examiner who was also exposed to the real-time feedback kept monitoring the pitch and loudness levels to ensure each participant had properly completed each task. All sEMG signals from the four sensors were recorded to four separate channels using the Delsys Trigno™ Wireless System (16-channel, Delsys, Natick).

### 2.3 Data analysis

All sEMG signals were analyzed using the EMGworks Analysis software (Delsys, Natick). To minimize the effects of the noise signals, a band-pass Butterworth filter between 20 and 500 Hz was used to filter all sEMG signals. The middle 2 s of each phonation (audio and sEMG signal) was extracted. A randomly selected 30% of the audio signals were perceptually evaluated by a certificated speech-language pathologist (SLP) to assess the stability of voice production (202 of the 209 tokens were evaluated as stable phonation), and all the extracted sEMG signals were analyzed for the root-mean-square (RMS). The RMS has been considered a reliable estimator that reflects the overall amplitude of sEMG, which would increase generally in relation to stronger muscle activation and force (De Luca, 1997; Stepp, 2012). The RMS value of each sEMG signal was normalized by the MVC of the corresponding sensor to minimize the effects of differences in submental fat among individuals (Netto and Burnett, 2006), resulting in an RMS %MVC value for each sEMG signal as the main outcome measure of this study.

To investigate the influencing factors on the RMS %MVC values of different muscles, the linear mixed-effects models were applied for calculating both fixed effects and random effects (Baayen et al., 2008) respectively in pitch manipulation and loudness manipulation tasks. In each condition, the gender (female, male), symmetry (left muscles, right muscles), vowel (/a/, /i/, and /u/), muscle (suprahyoid, SCM), and task (for pitch manipulation: baseline, +3, +6, −3, −6 semitones; for loudness manipulation: baseline, +5, +10, −5 dB) were set as fixed effects. Different participants were considered as the random effect. Analyses of the linear mixed-effects models were performed in R using the lme4 package (Bates et al., 2015). The *post hoc* comparisons were performed in R with the lsmeans package (Lenth, 2016). Different models were compared using likelihood ratio tests and the maximum likelihood identification predicted by the lowest Akaike Information Criterion (AIC, see Akaike, 1973). The goodness-of-fit of a certain model was represented by the R-square value (Johnson, 2014).

## 3 Results

There were 10 participants (5 males, 5 females) who failed to pass the screening criteria (VHI >11: six subjects; average auditory-perceptual rating score >2: three subjects; both criteria: one subject). As a result, 29 participants (16 females, 13 males) aged between 20 and 53 years (*M* = 28.2 years, *SD* = 7.7 years) were finally selected as qualified participants for this study. Table 1 listed the demographic and voice-related characteristics of the 29 participants. No significant gender difference was found for the VHI-10 (Mann-Whitney *U* = 0.728, *p* = 0.475) or the average auditory-perceptual rating score (Mann-Whitney *U* = 0.229, *p* = 0.846).

**TABLE 1:** Demographic and voice-related characteristics of the 29 participants.

Variables	Females (*n* = 16)		Males (*n* = 13)	
	Mean (SD)	[Minimum, maximum]	Mean (SD)	[Minimum, maximum]
Age (years)	26.6 (6.4)	[20, 47]	30.2 (8.6)	[20, 53]
VHI-10 (out of 40)	5.2 (4.2)	[0, 11]	6.0 (3.3)	[1, 10]
Perceptual Rating Score (out of 10)	0.7 (0.7)	[0, 2]	0.7 (0.5)	[0, 1.5]

VHI, voice handicap index; SD, standard deviation.

### 3.1 Muscle activities in pitch control

The mean and standard deviation of the RMS %MVC of sEMG signals at different pitch levels were listed in Table 2. Compared with the null model with only random intercepts of the subject, an addition of the by-subject random slopes for different muscles could significantly improve the model (*χ* ^*2*^ (2) = 1148.5, *p* < 0.001, AIC = 12,110). Random intercepts of the subject accounted for different baselines of the RMS %MVC among individuals. The by-subject random slopes for different muscles indicated that the effects of the fixed explanatory variables on the RMS %MVC values of the suprahyoid and SCM muscles were inconsistent among different participants.

**TABLE 2:** Mean (SD) RMS %MVC of sEMG signals at different pitch levels.

Muscle	Vowel	Pitch levels				
		Baseline	+3 semitones	+6 semitones	−3 semitones	−6 semitones
Left Suprahyoid	a	22.81 (14.70)	21.39 (17.16)	21.26 (15.38)	18.34 (16.67)	17.04 (15.50)
	i	28.98 (17.52)	27.90 (20.83)	29.39 (23.30)	22.96 (20.53)	20.17 (15.62)
	u	18.63 (15.44)	19.94 (19.12)	20.69 (19.37)	18.70 (19.96)	18.37 (18.47)
Right Suprahyoid	a	41.95 (25.39)	37.68 (26.90)	36.44 (24.81)	31.46 (22.61)	30.60 (22.95)
	i	43.86 (25.24)	41.31 (25.83)	43.42 (27.97)	36.18 (25.26)	32.46 (19.73)
	u	37.93 (27.03)	37.12 (28.90)	35.81 (28.14)	31.04 (26.96)	31.40 (21.34)
Left SCM	a	7.93 (7.38)	7.59 (5.56)	8.01 (5.94)	6.25 (5.11)	7.75 (7.53)
	i	7.50 (4.68)	7.76 (6.30)	8.91 (7.45)	8.25 (8.60)	8.73 (7.60)
	u	5.49 (3.04)	6.11 (3.75)	8.46 (7.25)	8.35 (11.91)	9.44 (9.08)
Right SCM	a	9.55 (7.47)	9.68 (7.33)	9.12 (6.28)	8.43 (8.01)	8.21 (6.41)
	i	10.82 (8.73)	9.75 (7.50)	9.98 (6.51)	10.11 (10.23)	9.84 (8.57)
	u	8.65 (7.92)	7.75 (5.85)	7.88 (6.13)	8.87 (8.33)	9.77 (10.79)

SD, standard deviation; RMS, root-mean-square; MVC, maximum voluntary contraction; SCM, sternocleidomastoid muscles.

**FIGURE 3:**
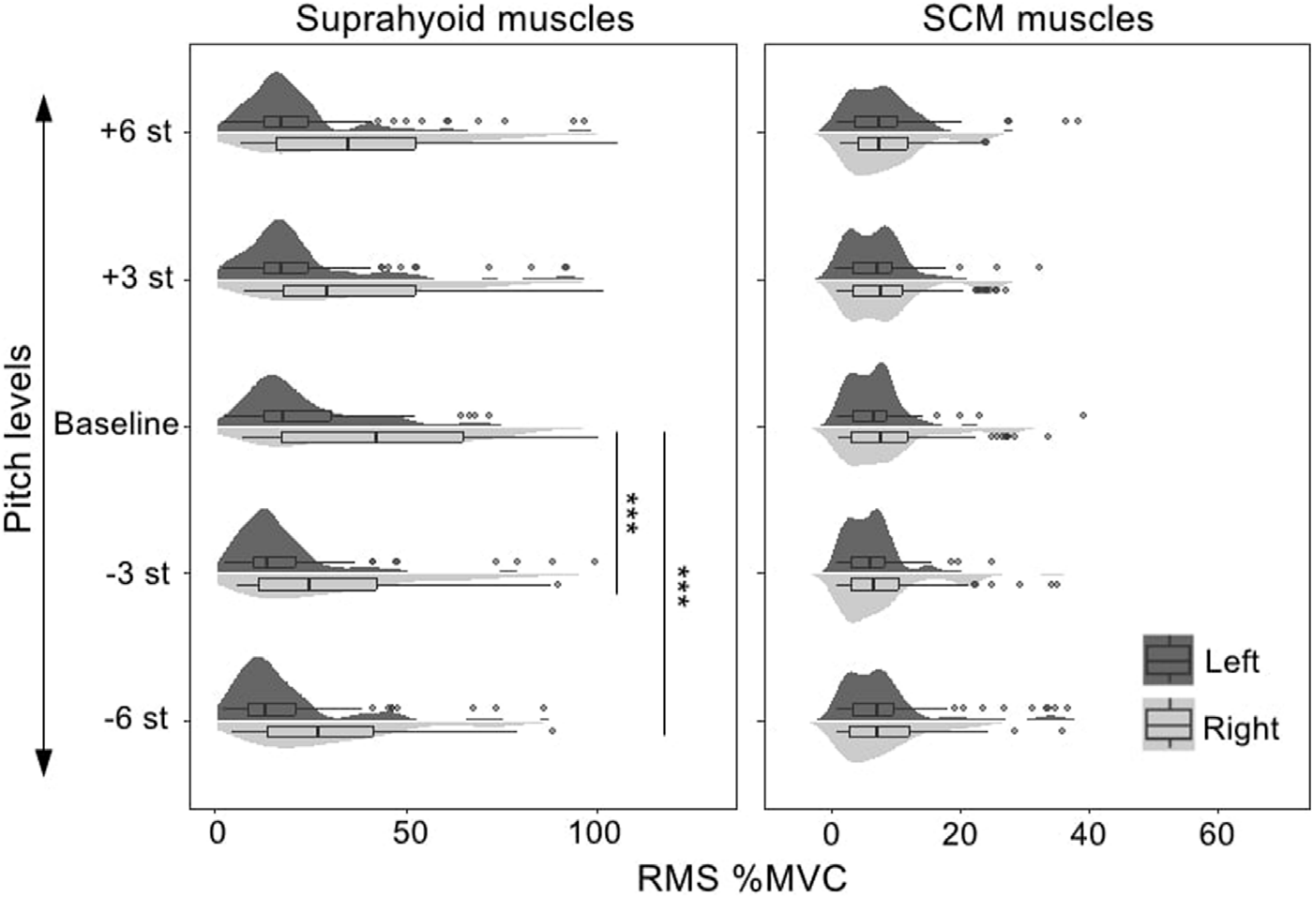
RMS %MVC value of the sEMG signals collected from the bilateral suprahyoid and SCM muscles during phonations at different pitch levels. SCM, sternocleidomastoid, st, semitones, “***”, *post hoc* contrast with *p*-values less than 0.001.

The best-fitted model (AIC = 11,654.9, *R* ^2^ = 0.75) contained muscle (suprahyoid, SCM), symmetry (left muscles, right muscles), vowel (/a/, /i/, and /u/), and task (baseline, +3, +6, −3, −6 semitones) as fixed factors, as well as the interactions of muscle*symmetry, muscle*vowel, and muscle*task. Given the interactions in the best-fitted model, *post hoc* comparisons of the fixed factors were performed to compare the contrasts in different conditions (see Table 3; Figure 3). A significant bilateral asymmetry in muscle activation at different pitch levels was found in both the suprahyoid and SCM muscles, with the right side demonstrating a higher RMS %MVC than the left side (*β* = 15.50, *p* < 0.001). The vowel /i/ had a significantly higher RMS for the suprahyoid muscles than the vowel /a/ (*β* = 4.81, *p* < 0.001) and /u/ (*β* = 5.67, *p* < 0.001), while /a/ and /u/ showed no difference in the use of suprahyoid muscles. Compared with the normal pitch baseline, the higher pitch changes, regardless of an increase by either 3 or 6 semitones, did not result in a significant change of the RMS %MVC for the suprahyoid muscles. Instead, the suprahyoid muscles demonstrated a significant decrease in RMS %MVC when lowering the pitch by 3 semitones (*β* = 5.76, *p* < 0.001) and 6 semitones (*β* = 7.20, *p* < 0.001; no significant difference between −3 semitones and −6 semitones). The SCM muscles, however, were not sensitive to different vowels and did not show any significant difference in RMS %MVC at different pitch levels.

**TABLE 3:** Post-hoc comparisons of the fixed factors in the fitted Linear mixed-effects model (in pitch control).

Fixed factors	Muscle = suprahyoid					Muscle = SCM				
	Estimate	SE	95% CI	*t*	*p*	Estimate	SE	95% CI	*t*	*p*
**Symmetry**										
Right—Left	15.50	0.76	[14.02, 16.98]	20.52	**<.0001**	1.45	0.66	[0.16, 2.75]	2.21	**0.0275**
**Vowel**										
a—i	−4.81	0.90	[-6.93, −2.69]	−5.33	**<.0001**	−0.92	0.81	[-2.81, 0.97]	−1.14	0.4887
a—u	0.86	0.91	[-1.27, −2.98]	0.95	0.6109	0.16	0.81	[-1.74, 2.05]	0.19	0.9797
i—u	5.67	0.91	[3.54, 7.79]	6.25	**<.0001**	1.07	0.81	[-0.82, 2.96]	1.33	0.3768
**Task**										
baseline—(+3 st)	1.38	1.17	[-1.82, 4.58]	1.18	0.7638	0.25	1.04	[-2.60, 3.09]	0.24	0.9993
baseline—(+6 st)	0.90	1.17	[-2.3, 4.11]	0.77	0.9388	−0.38	1.04	[-3.22, 2.46]	−0.37	0.9962
baseline—(−3 st)	5.76	1.17	[2.56, 8.95]	4.92	**<.0001**	−0.03	1.04	[-2.88, 2.81]	−0.03	1.0000
baseline—(−6 st)	7.20	1.17	[4.01, 10.40]	6.15	**<.0001**	−0.61	1.04	[-3.46, 2.23]	−0.59	0.9770
(−3 st)—(−6 st)	1.44	1.17	[-1.74, 4.63]	1.24	0.7289	−0.58	1.04	[-3.41, 2.26]	−0.56	0.9810

SCM, sternocleidomastoid muscles; SE, standard error; CI, confidential interval; st, semitone.

The *p* values were adjusted using the tukey method when there were more than 2 estimates. (bold means *p*-value < 0.05).

### 3.2 Muscle activities in loudness control

Table 4 demonstrated the mean and standard deviation of the RMS %MVC of sEMG signals at different loudness levels. Results of the Linear mixed-effects models also supported that adding the by-subject random slopes for different muscles would improve the null model with only random intercepts of the subject (*χ* ^*2*^ (2) = 679.75, *p* < 0.001, AIC = 10,059).

**TABLE 4:** Mean (SD) RMS %MVC of sEMG signals at different loudness levels.

Muscle	Vowel	Loudness levels			
		Baseline	+5 dB	+10 dB	−5 dB
Left Suprahyoid	a	22.81 (14.70)	18.37 (11.81)	27.85 (20.35)	13.00 (10.81)
	i	28.98 (17.52)	33.27 (21.08)	36.70 (25.39)	16.45 (10.79)
	u	18.63 (15.44)	15.97 (12.27)	19.31 (12.39)	13.17 (10.18)
Right Suprahyoid	a	41.95 (25.39)	31.85 (21.99)	44.86 (33.31)	32.55 (28.03)
	i	43.86 (25.24)	41.45 (22.02)	48.92 (33.05)	32.87 (21.37)
	u	37.93 (27.03)	33.58 (22.04)	37.75 (25.26)	32.81 (24.55)
Left SCM	a	7.93 (7.38)	8.75 (11.78)	11.66 (13.84)	6.99 (11.33)
	i	7.50 (4.68)	11.83 (14.60)	12.53 (14.97)	7.55 (11.13)
	u	5.49 (3.04)	8.68 (12.76)	10.81 (13.84)	6.45 (11.04)
Right SCM	a	9.55 (7.47)	8.11 (5.86)	9.09 (6.93)	6.01 (4.97)
	i	10.82 (8.73)	9.90 (7.13)	11.29 (7.30)	6.69 (6.30)
	u	8.65 (7.92)	6.56 (5.29)	6.88 (4.93)	5.67 (4.54)

SD, standard deviation; RMS, root-mean-square; MVC, maximum voluntary contraction; SCM, sternocleidomastoid muscles.

**FIGURE 4:**
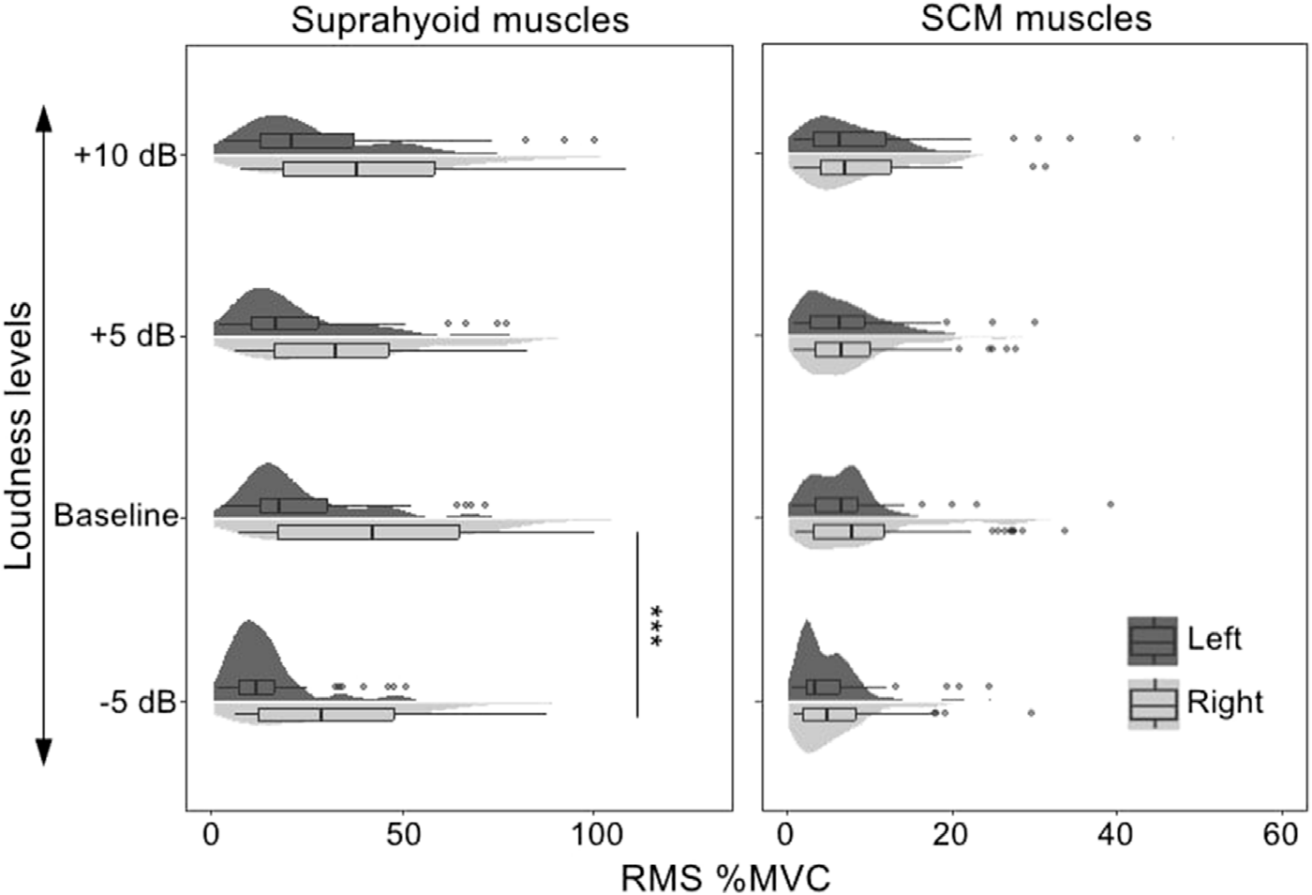
RMS %MVC value of the sEMG signals collected from the bilateral suprahyoid and SCM muscles during phonations at different loudness levels. SCM, sternocleidomastoid, “***”, *post hoc* contrast with *p*-values less than 0.001.

The fixed factors in the best-fitted model (AIC = 9694.5, *R* ^2^ = 0.67) also included muscle (suprahyoid, SCM), symmetry (left muscles, right muscles), vowel (/a/, /i/, and /u/), task (baseline, +5, +10, −5 dB), along with the interactions of muscle*symmetry, muscle*vowel, and muscle*task. The *post hoc* comparisons of the fixed factors were shown in Table 5; Figure 4. A significantly higher RMS %MVC for the right suprahyoid muscles (*β* = 16.33, *p* < 0.001) was also found during loudness manipulation, but the SCM muscles showed a bilaterally symmetric performance. The vowel /i/ had the highest RMS %MVC than /a/ (*β* = 6.35, *p* < 0.001) and /u/ (*β* = 9.26, *p* < 0.001) for the suprahyoid muscles, and /u/ elicited an even lower RMS %MVC amplitude than the vowel /a/ (*β* = 2.91, *p* = 0.0447). The suprahyoid muscles had a significantly declined RMS %MVC amplitude during a decreased (−5 dB) loudness manipulation (*β* = 9.44, *p* < 0.001), but no significant change was found during the increase of loudness. Consistent with the cases during pitch manipulations, the SCM muscle did not present a significant variation among different vowels or different loudness levels.

**TABLE 5:** Post-hoc comparisons of the fixed factors in the fitted Linear mixed-effects model (in loudness control).

Fixed factors	Muscle = suprahyoid					Muscle = SCM				
	Estimate	SE	95% CI	*t*	*p*	Estimate	SE	95% CI	*t*	*p*
**Symmetry**										
Right—Left	16.33	1.02	[14.34, 18.33]	16.05	**<.0001**	−0.60	0.88	[-2.32, 1.12]	−0.68	0.4952
**Vowel**										
a—i	−6.35	1.21	[-9.19, −3.51]	−5.24	**<.0001**	−1.24	1.08	[-3.76, 1.28]	−1.15	0.4821
a—u	2.91	1.22	[0.05, 5.77]	2.39	**0.0447**	0.16	1.08	[-1.45, 3.60]	1.00	0.5755
i—u	9.26	1.22	[6.41, 12.11]	7.62	**<.0001**	1.07	1.08	[-0.21, 4.85]	2.15	0.0801
**Task**										
baseline—(+5 dB)	3.554	1.40	[-0.05, 7.16]	2.54	0.0551	−0.61	1.24	[-3.80, 2.60]	−0.49	0.9621
baseline—(+10 dB)	−3.07	1.40	[-6.69, 0.54]	−2.19	0.1271	−1.99	1.25	[-5.20, 1.21]	−1.60	0.3791
baseline—(−5 dB)	9.44	1.40	[5.82, 13.05]	6.72	**<.0001**	1.79	1.24	[-1.41, 4.98]	1.44	0.4759

SCM, sternocleidomastoid muscles; SE, standard error; CI, confidential interval.

The *p* values were adjusted using the tukey method when there were more than 2 estimates. (bold means *p*-value < 0.05).

## 4 Discussion

This study sought to explore the contributions of the suprahyoid and SCM muscles to pitch and loudness control during phonation. The findings showed that when compared with the normal baseline, the suprahyoid muscles had significantly lower RMS %MVC amplitude during the production of lower pitches (−3 and −6 semitones) and loudness (−5 dB), which suggested relatively lower activations of the suprahyoid muscles in response to the lowering of pitch and loudness. However, no significant change was found with reference to the normal baseline in the activities of suprahyoid muscles when phonating with higher pitch (+3 and +6 semitones) and loudness (+5 and +10 dB). The SCM muscles were not contributing to pitch and loudness control during phonation.

It should be noted that the suprahyoid muscles did not show significantly different activity at the higher pitch levels (+3 and +6 semitones) when compared to the baseline, which seems to be inconsistent with what has been known that the suprahyoid muscles could serve as the elevator to help lift the larynx and result in a rise in pitch (e.g., Honda et al., 1999). As mentioned earlier, the 15 studies reviewed by Vilkman et al. (1996) also showed varied activation patterns of both the suprahyoid and infrahyoid muscles in response to pitch manipulations. The findings from these studies and that of the present study undoubtedly support the hypothesis of redundancy in the control of speech production, in which the human muscle system allows a high degree of freedom or flexibility in the muscle activation patterns which ultimately achieves the same target by using different muscles or different degrees of activation (Sorokin, 2010). In particular, the findings from the present study supported the hypothesis that the activation of the suprahyoid muscles is a sufficient but unnecessary condition for pitch rising, i.e., the pitch might rise following the activation of the suprahyoid muscles, but a higher pitch (within 6 semitones above the baseline, according to the present findings of this study) does not necessarily need more activation of the suprahyoid muscles. On the other hand, a lower activation of the suprahyoid muscles at the lower pitch levels (within 6 semitones below the baseline) was significant, which was consistent with the findings by Andersen and Sonninen (1960) that the mylohyoid muscles (suprahyoid muscle) showed less activation when singing in low pitch. Our findings suggest that the control of lower pitch does not necessarily rely on the suprahyoid muscles.

The suprahyoid muscles played a similar role in loudness control. Hirano et al. (1969) pointed out that changes in loudness were assisted by the tension and compression of the vocal cords, and the airflow through the glottis with different subglottic pressure. These locations are further away from the suprahyoid muscles than the infrahyoid muscles which have been discovered to activate during a stronger loudness (Hirano et al., 1967). It is therefore reasonable to see that the suprahyoid muscles did not show a different activation at the increased loudness levels. The suprahyoid muscles were even less activated at a weaker loudness level, which might indicate more uses of other muscles. This needs to be further investigated in future studies.

The present findings also revealed different activity levels of the suprahyoid muscles for different vowels. The largest activity level of the suprahyoid muscles was observed during the phonation of the vowel /i/ at different pitch and loudness levels. This was expected because the production of the vowel /i/ requires a forward pull of the tongue while drawing the hyoid bone upwards and forwards, which no doubt requires the assistance of the suprahyoid muscles such as the genioglossus and hyoglossus muscles, leading to the activity level of the suprahyoid muscles. It is therefore necessary to take caution when comparing the sEMG signals under different vowel phonation tasks.

This study found the SCM muscles insensitive to different vowels at all pitch and loudness levels. Although there were studies claiming that the SCM muscles might contribute to human speech and voice (Pettersen et al., 2005; Yu et al., 2016), some confounding factors in these previous studies might have affected the activity of the SCM muscles. Pettersen et al. (2005) focused on the SCM activation during respiratory phasing, while Yu et al. (2016) used natural Cantonese materials which could be affected by the laryngeal manipulations in meaningful speech. Given the fact that the SCM muscles are in the peri-laryngeal area but neither of their two joints is connected to a certain laryngeal structure, there is little justification to hypothesize that the SCM muscles would play an important role in pitch and loudness control. Although the SCM muscles showed relatively low level of activities as the tasks required non-extraneous respiratory effort, the influence of the adjacent scalene muscle, especially during the respiratory tasks (Chiti et al., 2008), could not be completely excluded. Hence, future studies should take cautions in conducting sEMG measurements of the SCM muscles using tasks that require respiratory effort. Considering the adequate size and location of the SCM muscles, we would like to propose that the SCM muscles could be used as a reference site for calibration or normalization of the sEMG signals collected from the anterior neck area during phonation tasks without excessive respiratory effort.

An interesting finding of the present study was the asymmetry of the bilateral sEMG signals for the suprahyoid muscles and the SCM muscles. Significantly higher RMS %MVC values were found for the suprahyoid (during pitch and loudness manipulations) and SCM (during pitch manipulations) muscles. It is intuitive to expect that the vocally healthy population would have a bilaterally symmetrical activity of the peri-laryngeal muscles. Indeed, some studies have reported a symmetrical activity of the bilateral suprahyoid and SCM muscles (Van Houtte et al., 2013; Lu et al., 2021). However, Hocevar-Boltezar et al. (1998) reported that three of the five subjects in their study showed different left and right EMG levels. Another interesting finding about the symmetry of muscle activity came from the study by Van Houtte et al. (2013). Although they did not find a bilateral difference in the RMS %MVC of the suprahyoid, infrahyoid, and SCM muscles in either the control group or patients with muscle tension dysphonia (MTD), a consistent right-dominant tendency existed in the increase of muscle activity during different phonation tasks. Compared to the reference of the resting condition, the right suprahyoid and SCM muscles had consistently higher activities than their left counterpart. Such right-dominant difference, surprisingly, was not consistently present in all the subjects in the MTD group. As the muscle asymmetry was not uncommon in populations with normal muscle function (e.g., the paraspinal muscle, see Niemeläinen et al., 2011), and all the participants in the present study were right-handed, this study would argue a possibility of the asymmetry of the bilateral suprahyoid and SCM muscles in the vocally-healthy group. It should be noted that personal variations were found in sEMG activity of the bilateral suprahyoid and SCM muscles (see Figures 3, 4). Furthermore, the sEMG amplitude does not typically show a linear relationship with the muscle activation and force (Disselhorst-Klug et al., 2009). Therefore, the identification of bilateral asymmetry of the muscle activities cannot be determined merely by the average RMS %MVC value presented in Tables 2, 4. Further determination of bilateral asymmetry would benefit from more advanced sEMG technology such as high-density sEMG (Zhu et al., 2019), and more studies are in need to explore the asymmetry of bilateral perilaryngeal muscles in the normal and voice-disordered groups.

## 5 Conclusion

The suprahyoid muscles demonstrated significantly fewer activities at the lower pitch (−3 and −6 semitones) and loudness (−5 dB) levels. The vowel /i/ was associated with relatively higher activities of the suprahyoid muscles than /a/ and /u/. The SCM muscles showed relatively little changes in producing different vowels and did not play a significant role in pitch and loudness control. Therefore, it is proposed that the SCM muscle could have the potential of being used as a reference for calibration and normalization of sEMG signals for the peri-laryngeal muscle involving phonatory tasks. A right-dominant bilateral asymmetry was found in the suprahyoid and SCM muscles, which would require further corroboration with more evidence.

## Data Availability

The original contributions presented in the study are included in the article/Supplementary Material, further inquiries can be directed to the corresponding author.
