# Supplementary material for: Surface electromyographic (sEMG) activity of the suprahyoid and sternocleidomastoid muscles in pitch and loudness control
Source: Front Physiol. 2023 May 4;14:1147795. doi: 10.3389/fphys.2023.1147795 (PMC10194839; doi:10.3389/fphys.2023.1147795)
Supplement: Supplementary file 1 [file Table1.DOCX]

Supplementary Material

Surface electromyographic (sEMG) activity of the suprahyoid and sternocleidomastoid muscles in pitch and loudness control

Feifan Wang*, Edwin M.-L. Yiu,

*** Correspondence:** Feifan Wang: feifan.wang@hku.hk

# Transcriptions of the Chinese poetry *Jingye Si*

The Hong Kong Cantonese pronunciation (Zee, 1991) of the Chinese poetry *Jingye Si* was transcribed with the International Phonetic Alphabet (IPA) as follows, along with the English translation (Wikipedia contributors, 2022):

IPA: tsʰɔŋ21 tsʰin21 mɪŋ21 jyt2 kwɔŋ5

English translation: Before my bed lies a pool of moonlight.

IPA: ji21 si2 tei2 sœŋ2 sœŋ5

English translation: I could imagine that it's frost on the ground.

IPA: kɵy35 tʰɐu21 mɔŋ2 mɪŋ21 jyt2

English translation: I look up and see the bright shining moon.

IPA: tɐi5 tʰɐu21 si5 ku3 hœŋ5

English translation: Bowing my head I am thinking of home.

**REFERENCE**

Zee, E. (1991). Chinese (Hong Kong Cantonese). *Journal of the International Phonetic Association*, *21*(1), 46–48. https://doi.org/10.1017/S0025100300006058

Wikipedia contributors. (2022, November 30). *Quiet Night Thought*. Wikipedia. <https://en.wikipedia.org/wiki/Quiet_Night_Thought>
